# Sequence and organization of coelacanth neurohypophysial hormone genes: Evolutionary history of the vertebrate neurohypophysial hormone gene locus

**DOI:** 10.1186/1471-2148-8-93

**Published:** 2008-03-26

**Authors:** Pai-Chung Gwee, Chris T Amemiya, Sydney Brenner, Byrappa Venkatesh

**Affiliations:** 1 Institute of Molecular and Cell Biology, A*STAR (Agency for Science, Technology and Research), Biopolis, 138673, Singapore; 2 Benaroya Research Institute at Virginia Mason, Seattle, Washington 98101, USA

## Abstract

**Background:**

The mammalian neurohypophysial hormones, vasopressin and oxytocin are involved in osmoregulation and uterine smooth muscle contraction respectively. All jawed vertebrates contain at least one homolog each of vasopressin and oxytocin whereas jawless vertebrates contain a single neurohypophysial hormone called vasotocin. The vasopressin homolog in non-mammalian vertebrates is vasotocin; and the oxytocin homolog is mesotocin in non-eutherian tetrapods, mesotocin and [Phe^2^]mesotocin in lungfishes, and isotocin in ray-finned fishes. The genes encoding vasopressin and oxytocin genes are closely linked in the human and rodent genomes in a tail-to-tail orientation. In contrast, their pufferfish homologs (vasotocin and isotocin) are located on the same strand of DNA with isotocin gene located upstream of vasotocin gene separated by five genes, suggesting that this locus has experienced rearrangements in either mammalian or ray-finned fish lineage, or in both lineages. The coelacanths occupy a unique phylogenetic position close to the divergence of the mammalian and ray-finned fish lineages.

**Results:**

We have sequenced a coelacanth (*Latimeria menadoensis*) BAC clone encompassing the neurohypophysial hormone genes and investigated the evolutionary history of the vertebrate neurohypophysial hormone gene locus within a comparative genomics framework. The coelacanth contains vasotocin and mesotocin genes like non-mammalian tetrapods. The coelacanth genes are present on the same strand of DNA with no intervening genes, with the vasotocin gene located upstream of the mesotocin gene. Nucleotide sequences of the second exons of the two genes are under purifying selection implying a regulatory function. We have also analyzed the neurohypophysial hormone gene locus in the genomes of opossum, chicken and *Xenopus tropicalis*. The opossum contains two tandem copies of vasopressin and mesotocin genes. The vasotocin and mesotocin genes in chicken and *Xenopus*, and the vasopressin and mesotocin genes in opossum are linked tail-to-head similar to their orthologs in coelacanth and unlike their homologs in human and rodents.

**Conclusion:**

Our results indicate that the neurohypophysial hormone gene locus has experienced independent rearrangements in both placental mammals and teleost fishes. The coelacanth genome appears to be more stable than mammalian and teleost fish genomes. As such, it serves as a valuable outgroup for studying the evolution of mammalian and teleost fish genomes.

## Background

The mammalian neurohypophysial hormones, vasopressin and oxytocin belong to a superfamily of structurally and functionally related nonapeptides. Vasopressin is primarily involved in the regulation of osmotic balance and contraction of smooth muscle cells in arteries while oxytocin is mainly involved in parturition and lactation [1,2]. Members of this superfamily are widespread in the animal kingdom with hormones similar to vasopressin and oxytocin isolated from invertebrates such as arthropods, annelids, and mollusks [3-5]. Among vertebrates, all jawed vertebrates (gnathostomes) characterized to date contain at least one vasopressin-like hormone and one oxytocin-like hormone. By contrast, the primitive jawless vertebrates (the cyclostomes, represented by lampreys and hagfishes) contain a single hormone, the vasotocin, which is more similar to vasopressin than to oxytocin [6,7]. Vasotocin is also the vasopressin homolog in non-mammalian jawed vertebrates. However, the oxytocin homologs are much more diverse: it is isotocin in teleost fishes (e.g., fugu) and other ray-finned fishes (e.g., bichir), mesotocin in non-eutherian tetrapods, and mesotocin and [Phe^2^]mesotocin in lungfishes [8-10]. In cartilaginous fishes, besides oxytocin in the Pacific ratfish [11], six homologs of oxytocin, termed aspargtocin, valitocin, asvatocin, phasvatocin, phasitocin, and glumitocin have been identified [12-14] (Table 1).

**Table 1:** Neurohypophysial hormones in vertebrates

*Vasopressin homologs*		
Vasopressin	Cys-Tyr-Phe-Gln-Asn-Cys-Pro-Arg-Gly (NH2)	Mammals
[Lys^8^]vasopressin	Cys-Tyr-Phe-Gln-Asn-Cys-Pro-Lys-Gly (NH2)	Pigs and some marsupials
[Phe^2^]vasopressin	Cys-Phe-Phe-Gln-Asn-Cys-Pro-Arg-Gly (NH2)	Some marsupials
Vasotocin	Cys-Tyr-Ile-Gln-Asn-Cys-Pro-Arg-Gly (NH2)	Non-mammals
		

*Oxytocin homologs*		

Oxytocin	Cys-Tyr-Ile-Gln-Asn-Cys-Pro-Leu-Gly (NH2)	Mammals, some marsupials, platypus, ratfish (*H. colliei*)
Mesotocin	Cys-Tyr-Ile-Gln-Asn-Cys-Pro-Ile-Gly (NH2)	Some marsupials, non-eutherian tetrapods, some lungfishes
[Phe^2^]mesotocin	Cys-Phe-Ile-Gln-Asn-Cys-Pro-Ile-Gly (NH2)	Australian lungfish
Isotocin	Cys-Tyr-Ile-Ser-Asn-Cys-Pro-Ile-Gly (NH2)	Ray-finned fishes
Glumitocin	Cys-Tyr-Ile-Ser-Asn-Cys-Pro-Gln-Gly (NH2)	Skates
Valitocin	Cys-Tyr-Ile-Gln-Asn-Cys-Pro-Val-Gly (NH2)	Sharks (*Sq. acanthias*)
Aspargtocin	Cys-Tyr-Ile-Asn-Asn-Cys-Pro-Leu-Gly (NH2)	Sharks (*Sq. acanthias*)
Asvatocin	Cys-Tyr-Ile-Asn-Asn-Cys-Pro-Val-Gly (NH2)	Sharks (*Sc. canicula; T. scyllium*)
Phasitocin	Cys-Tyr-Phe-Asn-Asn-Cys-Pro-Ile-Gly (NH2)	Sharks (*T. scyllium*)
Phasvatocin	Cys-Tyr-Phe-Asn-Asn-Cys-Pro-Val-Gly (NH2)	Sharks (*Sc. canicula*)

Adapted from Gimpl and Fahrenholz [47], and Hyodo et al. [48].

**Figure 1:**
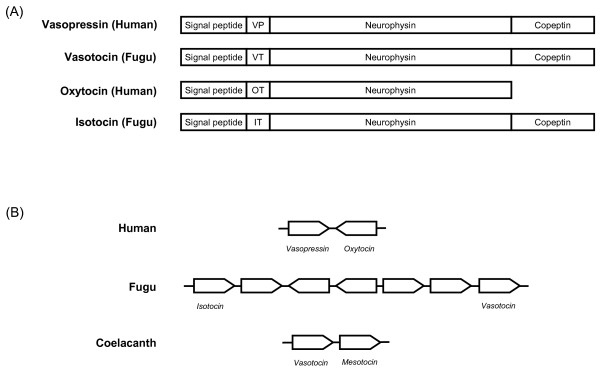
**The neurohypophysial hormones and genes in human, fugu and coelacanth**. (A) Structure of the neurohypophysial preprohormones. VP, vasopressin; VT, vasotocin; OT, oxytocin and IT, isotocin. (B) Organization of neurohypophysial hormone genes in human, fugu and coelacanth. Genes are represented by arrows. Only the neurohypophysial genes are labeled. The coelacanth genes were characterized in this study.

Molecular cloning of genes encoding neurohypophysial hormones has shown that the nonapeptides are synthesized as part of a larger precursor molecule that is composed of a signal peptide, the nonapeptide, and a neurophysin. The precursors of vasopressin-family hormones and the teleost fish isotocin hormone contain an additional molecule at the carboxyl terminal called copeptin (Fig 1A). The presence of a single gene encoding the vasotocin precursor in jawless vertebrates, and at least one gene each encoding a vasopressin-family precursor and an oxytocin-family precursor in jawed vertebrates, has led to the suggestion that the ancestral vasotocin gene has duplicated to give rise to vasopressin and oxytocin families of genes in jawed vertebrates. In human and rodents, the vasopressin and oxytocin genes are closely linked in a tail-to-tail orientation (i.e., located on the opposite strands of DNA), with intergenic regions ranging from 3 kb (mouse) to 12 kb (human) [15-17] (Fig 1B). Despite their close linkage, the two genes are regulated independently and are expressed in distinct populations of neurons in the supraoptic nuclei and paraventricular nuclei of the hypothalamus. Transgenic studies in mice and rats have indicated that the regulatory elements that mediate neuron-specific expression of vasopressin and oxytocin genes reside in the intergenic region of the two genes [18-20]. Interestingly, sequencing of the neurohypophysial hormone gene locus in the pufferfish (*Fugu rubripes*) showed that, in contrast to the tail-to-tail orientation of oxytocin and vasopressin genes in humans and rodent, pufferfish isotocin and vasotocin genes are located on the same strand of DNA, with vasotocin gene located downstream of isotocin gene, separated by five genes [21] (Fig 1B). These differences in the organization of human and pufferfish genes raise the question of whether the vasotocin and oxytocin homologs in the common ancestor of mammals and ray-finned fishes were linked like the human genes or the pufferfish genes. Sequencing of neurohypophysial hormone gene loci in vertebrates phylogenetically positioned close to the divergence of the mammalian and ray-finned fish lineages would shed light on the organization of neurohypophysial hormone genes in the last common ancestor of these vertebrates.

The lobe-finned fishes, lungfish and coelacanth, are the only two surviving lineages that arose between tetrapods and ray-finned fishes. The phylogenetic relationships of tetrapods to lungfish and coelacanth are unclear, although phylogenetic analyses of mitochondrial sequence and nuclear protein coding sequences, and a molecular marker (indel) seem to favor lungfish as the closest relative to tetrapods [22-26], and coelacanth as the most basal lobe-finned fish. It would, therefore, be interesting to determine the neurohypophysial hormones encoded by the coelacanth and the organization of neurohypophysial hormone genes in the coelacanth genome. Coelacanths were long believed to be extinct until a live specimen of the African coelacanth (*Latimeria chalumnae*) was caught off the coast of South Africa in 1938 [27]. A second species of coelacanth, *Latimeria menadoensis*, was caught more recently in Indonesia indicating that there are at least two living species of coelacanths [28,29]. Like cartilaginous fishes and lungfish, coelacanths store urea and trimethylamine oxide to stay hyperosmotic to seawater [30]. In this study, we have characterized the neurohypophysial hormone gene locus in the Indonesian coelacanth by isolating and sequencing a BAC clone. In addition, we have analyzed the neurohypophysial hormone gene loci in *Xenopus tropicalis*, chicken and gray short-tailed opossum (*Monodelphis domestica*) and investigated the evolutionary history of the vertebrate neurohypophysial hormone gene locus. Our results show that, in contrast to vasotocin and [Phe^2^]mesotocin in the Australian lungfish, coelacanth contains vasotocin and mesotocin similar to non-eutherian tetrapods, and that the arrangement of vasotocin and mesotocin genes in coelacanth is different from their homologs in both human and pufferfish.

## Results and Discussion

### Coelacanth neurohypophysial hormone gene locus

**Figure 2:**
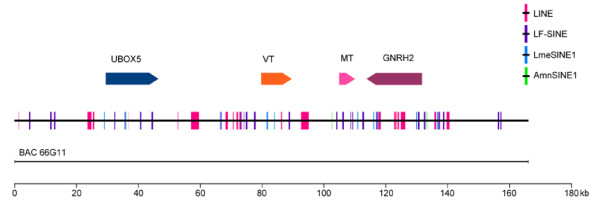
**The coelacanth neurohypophysial gene locus**. The BAC clone (66G11) sequenced is shown below. Arrows represent genes and indicate the direction of transcription. Vertical bars represent repetitive elements (LINE, LF-SINE, LmeSINE1 and AmnSINE1). VT, *vasotocin *gene; MT, *mesotocin *gene; Ubox5, *U-box domain containing 5*; Gnrh2, *gonadotropin-releasing hormone 2*.

By using a probe for the second exon of the coelacanth vasotocin gene, we isolated five overlapping BAC clones. One of the BACs, #66G11, was sequenced completely (the sequence data reported in this paper has been submitted to the GenBank database under accession number EU284132). The insert of this BAC is 166 kb long and contains both neurohypophysial hormone genes that are flanked by *Ubox5 *gene in the upstream and *Gnrh2 *gene in the downstream (Fig 2). The coelacanth neurohypophysial hormone genes encode vasotocin and mesotocin precursors like the non-mammalian tetrapods. It does not encode [Phe^2^]mesotocin present in the Australian lungfish. The coelacanth vasotocin and mesotocin genes each comprise three coding exons similar to the mammalian vasopressin and oxytocin genes and the pufferfish vasotocin and isotocin genes. However, the introns of coelacanth genes are considerably larger (1.55 kb to 5.57 kb) than their homologs in human (84 bp to 1.38 kb) and fugu (75 bp to 747 bp) mainly due to an abundance of repetitive sequences. Interestingly, the arrangement of coelacanth vasotocin and mesotocin genes is different from the arrangement of their homologs in both human and pufferfish. They are present on the same strand of DNA in a tail-to-head orientation with mesotocin gene located 15.4 kb downstream of vasotocin gene (Fig 2).

### Diverse SINE elements in the coelacanth neurohypophysial hormone gene locus

Approximately 17% of the 166-kb coelacanth neurohypophysial hormone gene locus sequence is represented by repetitive sequences, with LINEs and SINEs accounting for 7.9% and 6.9%, respectively (Fig 2). Altogether, this locus contains 18 LINE elements and 41 SINE elements. Previous studies have identified an ancient family of SINE elements in coelacanth, termed LF-SINE, which is specific to lobe-finned fishes [31]. The coelacanth genome is estimated to contain 10^5 ^copies of these SINEs, and some of these elements have been conserved in mammalian genomes as 'ultraconserved elements' (sequences more than 200 bp long and perfectly conserved in human, mouse and rat). Another family of SINE, called LmeSINE1, has also been identified in coelacanth [32]. Using the consensus sequence of the LmeSINEs, Nishihara et al [32], identified a novel family of SINEs which is widespread in the human and chicken genomes but absent in the coelacanth sequences that were available in the public domain. This family has been designated amniote-specific SINE1 or AmnSINE1 [32]. The SINE elements in the coelacanth neurohypophysial hormone gene locus include not only LF-SINEs and LmeSINE1s, but also two instances of AmnSINE1s (Fig 2, Additional file 1). The presence of AmnSINE1 in the coelacanth indicates that these elements are not specific to amniotes and are more ancient than previously thought.

### Coelacanth vasotocin and mesotocin precursors

**Figure 3:**
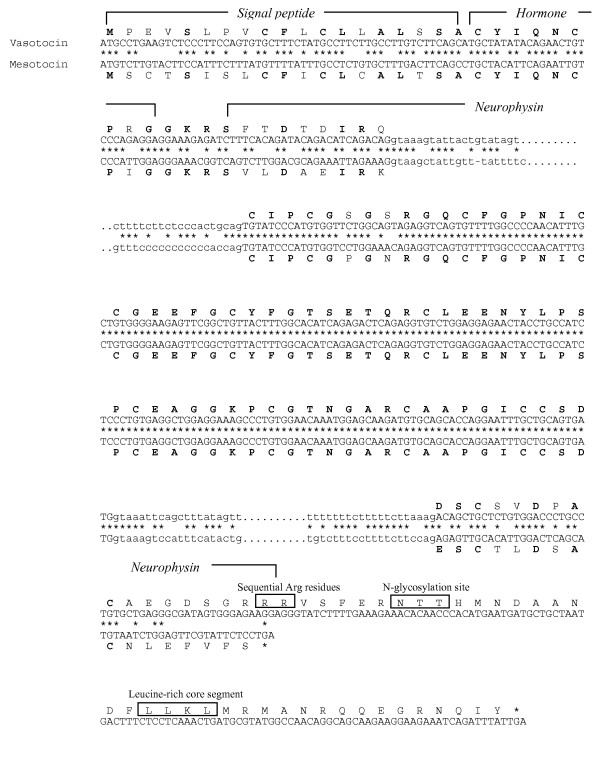
**Comparison of nucleotide and deduced amino acid sequences of coelacanth vasotocin and mesotocin genes**. Conserved nucleotides are indicated by an asterisk and identical amino acid residues are shown in bold font. Sequential Arg residues, N-glycosylation sites and Leu-rich core segment in the vasotocin precursor are boxed. The sequential Arg residues serve as a processing signal between the neurophysin and copeptin. Intronic sequences are shown in lower case.

**Figure 4:**
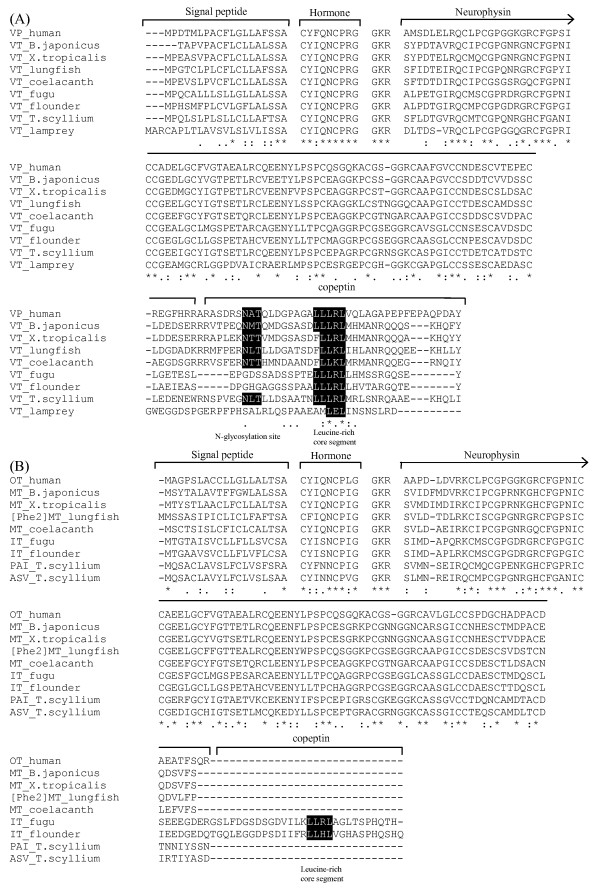
**Comparison of amino acid sequences of (A) vasopressin and (B) oxytocin family hormone precursors in vertebrates**. ClustalX was used to generate the alignment. Amino acid residues conserved in all vertebrates are marked with an asterisk. *B. japonicus*, *Bufo japonicus; X. tropicalis*, *Xenopus tropicalis*; and *T. scyllium*, *Triakis scyllium*. Accession numbers of sequences used in the alignment: NP_000481.2 (human VP), AAA48556.1 (*B. japonicus *VT), BAA24026.1 (lungfish VT), O42499 (fugu VT), BAA98140.1 (flounder VT), BAD27476.1 (*T. scyllium *VT) and BAA06669.1 (lamprey VT), NP_000906.1 (human OT), P08162 (*B. japonicus *MT), BAA24027.1 (lungfish [Phe^2^]MT), O42493 (fugu IT), BAA98141.1 (flounder IT), BAD27478.1 (*T. scyllium *phasitocin) and BAD27477.1 (*T. scyllium *asvatocin). Sequences for *Xenopus tropicalis *and coelacanth were generated in this study.

The coelacanth vasotocin gene encodes a 162-amino acid protein comprising a signal peptide, vasotocin, a neurophysin and a copeptin moiety (Fig 3). Vasotocin is linked to the neurophysin by a tripeptide sequence Gly-Lys-Arg which serves as a signal for proteolytic processing and carboxyl-terminal amidation of vasotocin (Fig 3). The two arginine residues at the end of the neurophysin (Figs 3 and 4A) are likely to serve as a processing signal between the neurophysin and the copeptin. All the cysteine residues that are considered important for the conformation of neurophysin are conserved in the coelacanth vasotocin neurophysin (Figs 3 and 4A). The copeptin includes a leucine-rich core segment like the copeptin of vasopressin-family precursors in other vertebrates, and an N-linked glycosylation site that is conserved in tetrapods, Australian lungfish and dogfish but absent in teleost fishes (Fig 4A).

The coelacanth mesotocin gene encodes a shorter protein of 124 amino acids comprising a signal peptide and mesotocin which is linked to a neurophysin by the tripeptide signal sequence, Gly-Lys-Arg (Fig 3). Like its homolog in the dogfish, Australian lungfish, toad and mammals, the coelacanth mesotocin precursor lacks a copeptin (Fig 4B). Thus, the teleost fish isotocin precursor is the only oxytocin-family precursor that contains a copeptin (Fig 4B). However, it should be noted that the teleost fish isotocin precursor does not contain an arginine residue between the neurophysin and the copeptin (Fig 4B), and consequently the copeptin moiety is not cleaved from the neurophysin molecule [33].

### Second exons of coelacanth vasotocin and mesotocin genes are under purifying selection

The second exons of the coelacanth vasotocin and mesotocin genes, which encode the central region of the neurophysin, exhibit an unusually high degree of identity at the amino acid (97.1%) as well as nucleotide level (98.0%). In fact, a stretch of 181 bp is perfectly conserved in the two genes (Fig 3). Previously, a similarly high level of nucleotide identity (197 bp perfectly identical) has been reported between the second exons of the bovine vasopressin and oxytocin genes [34]. In these genes, the high level of sequence identity extends into 135 bp of the preceding intron. This observation led to the suggestion that the two genes have experienced a recent gene conversion event [34]. We calculated the sequence identity between the second exons of vasopressin and oxytocin family genes from various vertebrates and found that they all exhibit a high identity (human, 92.5%; mouse, 95.5%; rat, 95%; bovine, 96.5%; chicken, 71.7%; opossum, 81.2%; *Xenopus*, 67.3% and fugu, 86.3%). However, besides the bovine genes, the high level of sequence identity extends into the preceding intron only in human genes (up to 28 bp) [34]. In coelacanth, the high level of sequence identity does not extend into the flanking introns (Fig 3), which suggests that, if coelacanth genes have indeed experienced gene conversion, it is restricted to the coding sequence. Gene conversion between coding regions of paralogous genes generally results in a high GC content at the third position (GC3) of codons [35-37]. To determine whether the coelacanth exons have experienced gene conversion, we calculated the GC3 content of all the three exons of the coelacanth vasotocin and mesotocin genes and compared them with GC3 content in bovine and human vasopressin and oxytocin genes. In the bovine and human genes, the GC3 content of the second exon is exceptionally high (94% to 95.5%). However, the GC3 content of their flanking exons is also high (87.5% to 98.2%) (Table 2), which indicates that either all three exons have experienced gene conversion or that these genes are located in a GC rich region of the genome. In the coelacanth vasotocin and mesotocin genes, the GC3 content of the second exons is rather low (41%) and comparable to that of the first and third exons (40% to 53%) (Table 2) which do not show a high level of sequence identity. Thus, the second exons of the coelacanth vasotocin and mesotocin genes do not seem to have experienced gene conversion. Alternatively, the high level of sequence identity could be the result of purifying selection acting on the nucleotide sequences of the second exons. Since the third positions of codons are also under constraint, it is possible that besides coding for amino acids, these exons may harbor sequences related to the stability of transcripts, microRNA targets [38] or regulatory elements such as exonic splicing enhancers [39] that require conservation at the nucleotide level.

**Table 2:** GC content of the third codon positions (GC3) in coding exons of neurohypophysial hormone genes

		Exon 1	Exon 2	Exon 3
Coelacanth	vasotocin gene	42.5%	41.2%	48.1%
	mesotocin gene	40.0%	41.2%	56.3%
Bovine	vasopressin gene	87.5%	94.0%	93.3%
	oxytocin gene	90.0%	95.5%	89.5%
Human	vasopressin gene	92.5%	95.5%	98.2%
	oxytocin gene	90.0%	94.0%	88.9%

The GC3 contents of all three exons of the coelacanth vasotocin and mesotocin genes are compared with the GC3 content in bovine and human vasopressin and oxytocin genes to determine if the coelacanth genes have undergone gene conversion.

### Evolutionary history of vertebrate neurohypophysial hormone gene locus

**Figure 5:**
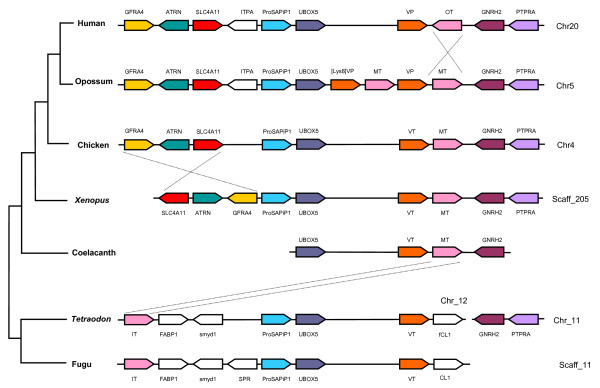
**Schematic diagram of neurohypophysial hormone gene locus in vertebrates**. Arrows represent genes and indicate the orientation of transcription. Genes flanking the *Tetraodon Gnrh2 *and *Ptpra *genes and the genes present downstream of fugu *CL1 *gene are not shown for the sake of clarity. Fugu orthologs for *Gnrh2 *and *Ptpra *genes are yet to be sequenced (likely to be present in the gaps of the genome assembly). GFRA4, *glial cell line-derived neurotrophic factor family receptor alpha 4 protei*n gene; ATRN, *attractin *gene; SLC4A11, *solute carrier family 4, sodium bicarbonate transporter-like, member 11*; ITPA, *Inosine triphosphate protein *gene; ProSAPiP1, *ProSAP-interacting protein 1 *gene; Ubox5, *U-box domain containing 5*; [Lys^8^]VP, *lysipressin *gene; VT, *vasotocin *gene; MT, *mesotocin *gene; OT, *oxytocin *gene; IT, *isotocin *gene, Gnrh2, *gonadotropin-releasing hormone 2*; PTPRA, *protein tyrosine phosphatase receptor type A *gene; FABP1, *fatty acid binding protein 1 *gene; smyd1, *SET and MYND domain containing 1 *gene; SPR, *sepiapterin reductase protein *gene; CCL1 *chemokine CL1 *gene.

To trace the evolutionary history of the neurohypophysial hormone gene locus, we analyzed vasotocin and mesotocin gene loci in the genome assembly of *Xenopus tropicalis*. The *Xenopus *genes for vasotocin and mesotocin are present on scaffold_205, which is ~1.85 Mb long. Their exon-intron organization is identical to that of their homologs in coelacanth, pufferfish and mammals (Additional files 2 and 3), and as expected, the precursors encoded by them are most similar to their orthologs in the toad and coelacanth (Fig 4). The *Xenopus *vasotocin and mesotocin genes are linked tail-to-head and flanked by *UBox5 *gene in the 5' region and *Gnrh2 *gene in the 3' region similar to their orthologs in coelacanth. Thus, the order and orientation of genes in this locus are highly conserved in *Xenopus *and coelacanth (Fig 5). The *Xenopus *neurohypophysial hormone gene locus is compact (~80 kb) compared to the coelacanth locus (~170 kb) mainly due to a paucity of repetitive sequences in the *Xenopus *locus. The conserved tail-to-head orientation of vasotocin and mesotocin genes in coelacanth and *Xenopus *indicate that the inverted orientation of oxytocin gene in human and rodents is the result of inversion of this gene in the tetrapod lineage after the divergence of amphibians. To determine more precisely when the inversion occurred, we analyzed neurohypophysial hormone gene locus in the genome assemblies of chicken and opossum. The vasotocin and mesotocin genes in the chicken genome are arranged similar to their orthologs in coelacanth and *Xenopus*. The opossum genome encodes vasopressin precursor similar to human and rodents but mesotocin precursor like non-eutherian tetrapods. Furthermore, it contains two copies of vasopressin and mesotocin genes due to a tandem segmental duplication, and one duplicated copy of the vasopressin gene codes for [Lys^8^]vasopressin precursor (Fig 5). Nevertheless, the two copies of the vasopressin and mesotocin genes are linked tail-to-head similar to their homologs in *Xenopus *and coelacanth. Thus, the inverted orientation of oxytocin gene in human and rodent is the result of a recent inversion event in placental mammals after they diverged from the marsupial lineage.

We also analyzed the neurohypophysial hormone gene locus in the genome of *Tetraodon nigroviridis*, a freshwater pufferfish. The isotocin and vasotocin genes in this fish are arranged similar to their orthologs in fugu and separated by four genes (Fig 5). Furthermore, the *Tetraodon Gnrh2 *and *Ptpra *genes whose orthologs are closely linked to mesotocin or oxytocin genes in tetrapods are located on a different chromosome in *Tetraodon *and most likely in fugu (Fig 5). Thus, the neurohypophysial hormone gene loci in pufferfishes appear to have experienced multiple rearrangements. As such, the arrangement of isotocin and vasotocin genes in pufferfishes is unlikely to represent the arrangement of their homologs in the last common ancestor of ray-finned fishes and lobe-finned fishes. The two neurohypophysial hormone genes in the common ancestor were more likely to have been linked closely in a tail-to-head orientation similar to their homologs in coelacanth, *Xenopus*, chicken and opossum. Sequencing of neurohypophysial hormone gene loci in cartilaginous fishes should confirm this hypothesis.

### Evolution of vasopressin and oxytocin families of hormones

**Figure 6:**
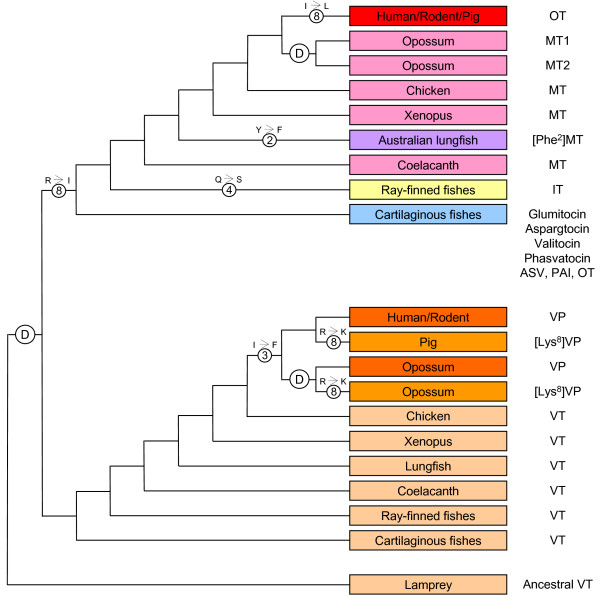
**Evolution of vasopressin and oxytocin family of nonapeptides**. The letter 'D' within the circle represents a gene duplication event. The numbers within the circles denote the position of the amino acid that has been substituted (shown above the circle). OT, oxytocin; MT, mesotocin; IT, isotocin; VP, vasopressin; [Lys^8^]VP, lysipressin, VT, vasotocin; ASV, asvatocin; PAI, phasitocin. Protein sequencing has shown that some lungfishes contain mesotocin [9, 10] and some marsupials contain [Phe^2^]vasopressin [46]. They are not shown for the sake of clarity.

The evolution of vasopressin and oxytocin family hormones is a classical example of the origin of a novel gene by the duplication of a gene. While vasopressin family peptides are basic peptides, the oxytocin family peptides are neutral peptides. These distinct chemical properties are conferred by the eighth residue of the nonapeptide which is Arg or Lys in vasopressin family, and Leu, Ile, Gln or Val in oxytocin family (Table 1). Following the duplication of the ancestral vasotocin gene, one copy has been conserved to encode the basic peptide, while the other copy has diverged to give rise to the novel neutral peptides of the oxytocin family (Fig 6). The identification of vasotocin gene in coelacanth and vasopressin gene in opossum indicates that vasotocin is highly conserved during evolution of vertebrates from cyclostomes to birds, and is replaced by vasopressin in mammals (Fig 6). The evolutionary stability of vasotocin in non-mammalian vertebrates probably reflects its fundamental role in maintaining osmotic balance in these vertebrates. In contrast to the highly conserved vasotocin in non-mammalian vertebrates, oxytocin family hormone exhibits wide diversity in these vertebrates. The most basal jawed vertebrates, the cartilaginous fishes, contain seven different types of oxytocin-like hormones, which suggests a relaxed pressure on this family of hormone owing presumably to the redundancy of the second hormone following the duplication of the ancestral vasotocin gene (Fig 6). While the ray-finned fishes contain isotocin, mesotocin is conserved in coelacanth, lungfish, and non-eutherian tetrapods. The Australian lungfish appears to be unique in possessing [Phe^2^]mesotocin. It is interesting that although ray-finned fishes are the largest and most diverse group of vertebrates, they posses only isotocin in contrast to cartilaginous fishes which contain seven different forms of oxytocin-like hormone. The presence of only isotocin in ray-finned fishes reflects a selective pressure on this hormone in this largest group of vertebrates.

## Conclusion

We have sequenced neurohypophysial hormone genes from coelacanth and shown that coelacanth contains vasotocin and mesotocin like the non-eutherian tetrapods. We also show that the coelacanth vasotocin and mesotocin genes, and their homologs in *Xenopus*, chicken and opossum are linked in tandem in a tail-to-head orientation unlike the tail-to-tail orientation of their homologs in placental mammals, and tail-to-head orientation of isotocin and vasotocin genes in pufferfishes. These results indicate that the neurohypophysial hormone gene locus has undergone independent rearrangements in placental mammals and teleost fishes. The analysis of the neurohypophysial hormone gene locus has shown that the coelacanth genome is likely to be more stable than that of ray-finned fishes and mammals. This underscores the importance of coelacanth as a valuable outgroup for tracing evolutionary changes in the tetrapod and ray-finned fish lineages.

## Methods

### PCR amplification of coelacanth vasotocin fragment

The amino acid sequences of vasotocin precursors from various vertebrates were aligned using ClustalX and several pairs of degenerate PCR primers were designed to amplify a fragment of vasotocin gene from coelacanth. Genomic DNA of the Indonesian coelacanth extracted from the gill tissue [40] was used as a template. The primer-pair, 5'-GGN CCN WAY ATH TGY TGY GG-3' and 5'-CAN AYN CCN GGN GCN GCR CA-3', corresponding to the conserved sequences in the neurophysin region (GPN/YICCG and CAAPGV/IC) of the Japanese toad (accession number P08163) and the Australian lungfish (accession number BAA24026) vasotocin precursors, was effective in amplifying a genomic fragment of the expected size (~160 bp). The PCR cycling conditions used consisted of an initial denaturation step at 95°C for 2 min, followed by 35 cycles of 95°C for 30 sec, 50°C for 1 min and 72°C for 30 sec, with a final elongation step at 72°C for 5 min. The PCR product was cloned into a T-vector and sequenced. BLASTX search of the sequence indicated that it is highly similar to neurophysin molecule of the vasotocin precursor cloned from other vertebrates. The sequence of the PCR fragment was extended by inverse PCR using libraries of circularized DNA as described before [40]. The extended sequence, a 2.8 kb *XmnI *fragment, includes the complete second exon and partial sequences for the flanking introns.

### Isolation and sequencing of BAC

A 283-bp PCR product (amplified using the primers 5'-CTG CAG TGT ATC CCA TGT GGT TCT GG-3' and 5'-GTA TCG CCC AAT CAC TAG-3') that includes the complete second exon and 72 bp of the succeeding intron was used to screen an Indonesian coelacanth BAC library [41], and five positive BAC clones (66G11, 72J19, 117H6, 159M19, and 252B6) were identified. Restriction fragment analysis indicated that the five BACs belong to the same locus. Since the probe used codes for the central portion of the neurophysin which is conserved across species as well as between vasopressin and oxytocin-like precursors, we believe that this is the only neurohypophysial hormone gene locus in coelacanth. One of the positive BAC clones, clone #66G11, was sequenced completely using the shotgun sequencing strategy. In brief, the shotgun sequencing strategy involved shearing of BAC DNA by ultrasonication followed by end-filling by Klenow treatment, and separation of the fragments on a 1% agarose gel. Fragments in the range of 2–3 kb were then extracted from the gel and subcloned into the *Eco*RV site of pBluescript SK vector. The plasmid inserts were sequenced using standard BigDye Terminator v3.1 chemistry on an ABI 3730xl DNA analyzer. Shotgun reads were assembled with SeqBuilder (Lasergene 6 software package, DNASTAR) and gaps were filled by 'primer-walking' using BAC DNA as a template or by sequencing PCR products.

### Sequence analysis

Protein coding genes were predicted based on homology to known proteins in the National Centre for Biotechnology Information database [42] and their exon-intron boundaries were refined by manual inspection. Multiple sequence alignments of protein sequences were carried out with ClustalX Version 1.83 [43]. Repetitive sequences were identified using the RepeatMasker (version open-3.1.6)[44]. The genomic sequences of the neurohypophysial hormone gene locus for human (March 2006 assembly), *Xenopus tropicalis *(assembly version 4.1), chicken (assembly version 2.1), gray short-tailed opossum (Jan 2006 assembly), fugu (assembly version 4.0) and *Tetraodon nigroviridis *(February 2004 assembly) were obtained from the UCSC Genome Browser [45]. The genes in the *Xenopus*, chicken and opossum neurohypophysial hormone gene locus were annotated based on homology to known protein sequences in NCBI database and the exon-intron boundaries were refined by manual verification.

## Authors' contributions

BV and SB conceived and designed the project. CTA performed screening and characterization of the BAC library. PG carried out sequencing, annotation and analysis of the sequences reported. PG and BV wrote the manuscript. All authors read and approved the final manuscript.

## Supplementary Material

Additional file 1 Alignment of nucleotide sequences of AmnSINE1 elements from coelacanth, human and chicken. Alignment of nucleotide sequences of two instances of AmnSINE1 elements from coelacanth [AmnSINE1a(Coe) and AmnSINE1b(Coe)] as well as AmnSINE1 elements from human and chicken [32]. Click here for file

Additional file 2 The neurohypophysial gene locus in *Xenopus tropicalis*. Arrows represent genes and indicate the direction of transcription. The sequence for this locus is downloaded from UCSC Genome Browser (assembly version 4.1)[45], and the genes were annotated based on homology to known protein sequences and the exon-intron boundaries were refined by manual annotation. VT, *vasotocin *gene; MT, *mesotocin *gene; Ubox5, *U-box domain containing 5 *gene; Gnrh2, *Gonadotropin-releasing hormone 2 *gene. Click here for file

Additional file 3 Comparison of nucleotide and deduced amino acid sequences of *Xenopus tropicalis *vasotocin and mesotocin genes. Conserved nucleotides are indicated by an asterisk and identical amino acid residues are shown in bold font. Sequential Arg residues, N-glycosylation sites and Leu-rich core segment in the vasotocin precursor are boxed. Click here for file
