# Supplementary material for: Sequence and organization of coelacanth neurohypophysial hormone genes: Evolutionary history of the vertebrate neurohypophysial hormone gene locus
Source: BMC Evol Biol. 2008 Mar 26;8:93. doi: 10.1186/1471-2148-8-93 (PMC2315648; doi:10.1186/1471-2148-8-93)
Supplement: Additional file 1 — Alignment of nucleotide sequences of AmnSINE1 elements from coelacanth, human and chicken. Alignment of nucleotide sequences of two instances of AmnSINE1 elements from coelacanth [AmnSINE1a(Coe) and AmnSINE1b(Coe)] as well as AmnSINE1 elements from human and chicken [32]. [file 1471-2148-8-93-S1.pdf]

```
AmnSINE1(Human)       ------GYCTRCAGC-CAYASCACCTCGGGCTR---TGATCTCGTCAGATCTCACAAGCT
AmnSINE1(Chicken)     -----AGCCTGYAGC-CATACCACCTCGGGCTG---TGATCTCGTCAGATCTCACAAGCT
AmnSINE1a(Coe)        ---CTGCTGCATCAC-CAGGCCAGCTTTGGCAAAAATGTTTTAGTTGTGTAGAATGAAAG
AmnSINE1b(Coe)        GAGGGGCAGTAATACTCAGAATACCCCTTGTTA---GGTTAAGTTCTTCCTGAGCCAAAA
                                    * **    * *    *       * *    *           *

AmnSINE1(Human)       AAGCAGGGTCGG-GCCTGGTCAATDCTTG--GATG--GAAGMCCTCCAAGGAAAAC---C
AmnSINE1(Chicken)     AAGCAGGGTCGG-GCCTGGTCARTACTTG--GATG--GAAGACCTCCAAGGAAAAC---C
AmnSINE1a(Coe)        AGATGCCATTAATATCTAAGCATTACTGTATGATGTTATAGTCTATTGATGATTATG--T
AmnSINE1b(Coe)        GGCTCTTGCTTA--TCTG-CTGTTTCTAT--GGTG---CAAGTGGACGAGAACTATGTTC
                                     **      * **    * **    *        *  *  *

AmnSINE1(Human)       CAGGTGCTRCAGGAAGTGGTGYT-GGTGATTCAGTAGGTGGCACTCTTCCCTCTGA-GTC
AmnSINE1(Chicken)     CAGGTGCTGYAGRAAGYGGTGYT-GGTGATTCAGTAGGTGGCACTCTTCCCTCTGA-GTC
AmnSINE1a(Coe)        CTCCTGTTTCCAACACCGAGGTTAGGTAGGGCCAACAACATCAGTTTCTTGACTGAAGTA
AmnSINE1b(Coe)        TCCCTGGATACGACACCAGTGTATTACAGG----------TTAACTCCCCAGC-------
                          **        *     *                     *         *

AmnSINE1(Human)       AGTACTGAACCAATGYCCCAGCATGGTGTTAGGGGGCACTGTGYTGCYGGAGGTGCCGTC
AmnSINE1(Chicken)     AGTACTGAACCAATGCCCCAGCATGGTGTTARRGGGCACTGTGTTGYTGGAGGTGCCGTC
AmnSINE1a(Coe)        AGTACCTAGACTGCCAGTCA-CCTCAAGTTTAGGGCCCCTACAGCACCGTCTGTGCTGTT
AmnSINE1b(Coe)        AACAGTCAGGTACTCATTCACAGTTGAGTTGACTGA-GGTGCACC-TCGTCTTCCCTGGC
                      *  *   *          **   *   ***    *    *        *      * *

AmnSINE1(Human)       --TTTCGGATGAGACGTAAAACCGAGGTCCTGACCACTTGCGGTCATTAAAGATCCCATG
AmnSINE1(Chicken)     --TTTCGGATGAGACGTAAAACCGAGGTCCTGACCACTTGCGGTCATTAAAGATCCCATG
AmnSINE1a(Coe)        GGTCTCCCATCAAA-GTACTAGTCAGG-CCTGACCCTGCTTAGCTTCTGAA-ATCAGACA
AmnSINE1b(Coe)        AGTCCCCCATCCAA-GTACCAACCAGG-CCTGGCCCTGCTTAGCTTCTGAG-AACAAACA
                        *  *  **   * ***  *   *** **** **       *    * *  * *  *

AmnSINE1(Human)       GCACTTTTCGTAAGAGTAGGGGTGTTAACCCCGGTGTCCTGGCCAAATTCCAATTCGGGT
AmnSINE1(Chicken)     GCACTTTTCGTAAGAGTARGGGTGTTAACYCCGGTGTCCTGGCCAAATTCCARYTCGGGT
AmnSINE1a(Coe)        GGATCAGGTGCACCCAGGGTGGGTTATAGTCCATTACTATCATTTAATGACCATTACCAT
AmnSINE1b(Coe)        GGATCGGGAACATCCA-AGCCAGTAAGGCACCTCCCTTGTTAGGTACTGGAAGATAATTT
                      * *        *                  **       *     * *      *    *

AmnSINE1(Human)       AATTACATTCTGCCTACCTAAATTCCCYCTGCAGT-TTCAATTGGATACGGTATTC-TTC
AmnSINE1(Chicken)     AATTACATTCTGCCTACCTAAATTCCCTCTGCAGT-TTCAATTGGATACGGTATTC-TTC
AmnSINE1a(Coe)        AG--ACATTTGACTTCC----ATCTGGTGCATAATACTGAGTTTGAGACAATTGCCACTC
AmnSINE1b(Coe)        -GCCACACTGTATATTA----TGTTTGTGGGTAAAAAAAAAAAAAAAAAAAAAAAA----
                          *** *     *                 *      *     * *

AmnSINE1(Human)       ACTTCCTGTCCTAA-ACTGTTGTGTAGTGTTGCTGTGCGCTGTTAAACAGCTGCCGCGTT
AmnSINE1(Chicken)     AYTTCCTGTCCTAA-ACTGTTGTGTAGTGTTGCTGTGCGCTGTTAAACAGCTGCCGCGTT
AmnSINE1a(Coe)        AATCTCAGTAGCACTGATGTCGC-CAGTATT-CTGCACATAAGTAAA-GGCAGGAGCAAG
AmnSINE1b(Coe)        AGATGTGGCAGCCT--TTTTTTTTCATTTATTTGTCATGCTTGTATGTGTCTTGATGTTA
                      *      *         * *     * *  *            **     *

AmnSINE1(Human)       YCACCCCAGAGGTGGCTGCATTTCAGTGGTGGGTGAARTGATCYCTATATGTAGCTTGTA
AmnSINE1(Chicken)     TCACCCCAGAGRTGGCTGCATTTCAGTGGTGGRTGAAATGATCYBTATATGTAGYTTGTA
AmnSINE1a(Coe)        GAGCAGTGGCTTCAGATGCAAAACAGGTGCCAGTCCCTTTTCTTTTTTAATTGGTTTTTC
AmnSINE1b(Coe)        ACTTATTATATTTAGTTG-AGTTTTATTGTGTGTATCTTGTAAACTTAAAATACAATCAA
                                    * ** *        *    *    *      *  *  *    *

AmnSINE1(Human)       AAGCGCTTTG-------GGATCCTTCGGGATGAAAGGCGCTATATAAAC-GTAAGGTATT
AmnSINE1(Chicken)     AAGCGCTTTG-------RGATCCTTCGGGATGAAAGGCGCTATATAAAC-GTAARGTATY
AmnSINE1a(Coe)        CAGTGCTCCACTTACCAGGATTGGTTCTTCCCAGCTCCGCTCAACTCTCTGTCTGGCACT
AmnSINE1b(Coe)        CAGTGAATTAAA----AAAATAAACAAAATGCAGAGAAAGGAAAGTTTC-TTACTGCAGT
                       ** *              **           *          *    *  *   * *

AmnSINE1(Human)       ATTATTATT-----------
AmnSINE1(Chicken)     ATTATA--------------
AmnSINE1a(Coe)        GTGGAACCTTGACTGGAGCT
AmnSINE1b(Coe)        GAACAAGCTAGATAACAA--
```
