# Supplementary figures and images for: Sequence and organization of coelacanth neurohypophysial hormone genes: Evolutionary history of the vertebrate neurohypophysial hormone gene locus
Source: BMC Evol Biol. 2008 Mar 26;8:93. doi: 10.1186/1471-2148-8-93 (PMC2315648; doi:10.1186/1471-2148-8-93)

UBOX5

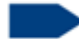

VT

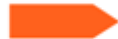

MT

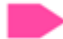

GNRH2

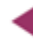

Scaff\_205

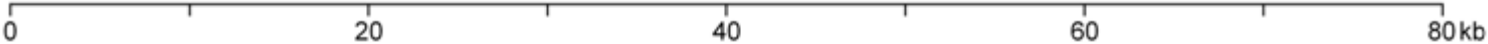

Supplement: Additional file 2 — The neurohypophysial gene locus in Xenopus tropicalis. Arrows represent genes and indicate the direction of transcription. The sequence for this locus is downloaded from UCSC Genome Browser (assembly version 4.1)[45], and the genes were annotated based on homology to known protein sequences and the exon-intron boundaries were refined by manual annotation. VT, vasotocin gene; MT, mesotocin gene; Ubox5, U-box domain containing 5 gene; Gnrh2, Gonadotropin-releasing hormone 2 gene. [file 1471-2148-8-93-S2.pdf]
