# Supplementary material for: Sequence and organization of coelacanth neurohypophysial hormone genes: Evolutionary history of the vertebrate neurohypophysial hormone gene locus
Source: BMC Evol Biol. 2008 Mar 26;8:93. doi: 10.1186/1471-2148-8-93 (PMC2315648; doi:10.1186/1471-2148-8-93)
Supplement: Additional file 3 — Comparison of nucleotide and deduced amino acid sequences of Xenopus tropicalis vasotocin and mesotocin genes. Conserved nucleotides are indicated by an asterisk and identical amino acid residues are shown in bold font. Sequential Arg residues, N-glycosylation sites and Leu-rich core segment in the vasotocin precursor are boxed. [file 1471-2148-8-93-S3.pdf]

```
                            Signal peptide                          Hormone

            M  P  E  A  S  V  P  A  C  F  L  C  L  L  A  L  S  S  A  C  Y  I  Q  N  C
Vasotocin  ATGCCTGAAGCTTCGGTCCCTGCTTGTTTCCTGTGCCTTCTTGCCCTCTCCTCAGCCTGCTACATCCAGAACTGC
           *** *  *     *  *  * ** *** ** * ******** ** *** * ***** *********** ******
Mesotocin  ATGACCTACAGCACCCTGGCCGCGTGTCTCTTCTGCCTTCTGGCTCTCACTTCAGCTTGCTACATCCAAAACTGC
            M  T  Y  S  T  L  A  A  C  L  F  C  L  L  A  L  T  S  A  C  Y  I  Q  N  C

                                                         Neurophysin

            P  R  G  G  K  R  S  Y  P  D  T     E  L  R  Q
           CCCAGAGGGGGCAAGAGGTCCTACCCCGACAC---AGAACTCCGACAGgtgcagtatcttttatcctt.......
           ****  ** ** ***** **       ****     **  ** *  ****           *  ***
           CCCATTGGAGGAAAGAGATCGGTGATGGACATCATGGACATCAGGAAGgtaagagtgtcgctgccctg.......
            P  I  G  G  K  R  S  V  M  D  I  M  D  I  R  K


                                   C  M  Q  C  G  P  G  N  R  G  N  C  F  G  P  N  I
           ...atcatgttgttcccttgcagTGCATGCAGTGTGGGCCGGGAAACAGAGGCAACTGCTTCGGGCCGAACATTT
                *         *  *  **** ** *  ********* **** * ***  * ** ** ***** ***** *
           ...tcccaacccaatctctttagTGTATCCCTTGTGGGCCGAGAAATAAAGGACATTGTTTTGGGCCAAACATCT
                                   C  I  P  C  G  P  R  N  K  G  H  C  F  G  P  N  I


           C  C  G  E  D  M  G  C  Y  I  G  T  P  E  T  L  R  C  V  E  E  N  F  V  P
           GCTGCGGAGAAGATATGGGGTGTTACATCGGCACCCCCGAGACCCTCCGCTGTGTAGAAGAGAACTTCGTGCCGT
           * ** ** ** **  **** ** *** * ** *** * *****  *  * **    ******** * * * ** *
           GTTGTGGGGAGGAACTGGGTTGCTACGTTGGGACCTCGGAGACGTTAAGGTGCCAGGAAGAGAATTACCTTCCTT
           C  C  G  E  E  L  G  C  Y  V  G  T  S  E  T  L  R  C  Q  E  E  N  Y  L  P


           S  P  C  E  A  G  G  R  P  C  S     T  G  G  R  C  A  A  P  G  I  C  C  N
           CCCCTTGTGAAGCCGGGGGGAGACCCTGCAG---TACAGGCGGAAGGTGTGCAGCCCCCGGAATCTGCTGCAATG
           * ** *****  * *** * * *** **  *    *  ** ****  ** ** *** * ** ***********
           CTCCATGTGAGTCGGGGAGAAAACCTTGTGGGAACAATGGAGGAAACTGCGCCGCCTCTGGCATCTGCTGCAATC
           S  P  C  E  S  G  R  K  P  C  G  N  N  G  G  N  C  A  A  S  G  I  C  C  N


           D                                                      E  S  C  S  L  D  S
           ACGgtaatgagcc-ccagtaaagc..........tttgtttcattgcatttgcagAGAGTTGCAGTTTGGACTCG
           ******* **    ** **   *                *   *  **** ******** ****   ****  **
           ACGgtaaggaatggcctgttctg-..........cccaatctgtatcatt-gcagAGAGCTGCACAGTGGATCCG
           H                                                      E  S  C  T  V  D  P
                Neurophysin

                          Sequential Arg residues          N-glycosylation site

            A  C  L  D  D  E  S  E  R  R  R  A  P  L  E  K  N  T  T  V  M  D  G  S
           GCCTGCCTGGACGACGAGAGCGAAAGGCGGCGGGCGCCACTGGAAAAGAACACGACCGTCATGGACGGCTCAG
           *****   * * ***     **  *     *  *
           GCCTGTGAGCAGGACT----CGGTATTTTCCTAG---------------------------------------
            A  C  E  Q  D       S  V  F  S  *


                   Leucine-rich core segment

           A  S  D  F  L  L  R  L  M  H  M  A  N  R  Q  Q  Q  A  K  H  Q  Y  Y  *
           CCAGCGACTTTCTCCTGCGGCTTATGCACATGGCTAACAGACAACAGCAAGCCAAGCACCAATACTACTGA
           -----------------------------------------------------------------------
```
